# Growth Factors Released from Gelatin Hydrogel Microspheres Increase New Neurons in the Adult Mouse Brain

**DOI:** 10.1155/2012/915160

**Published:** 2012-10-10

**Authors:** Kanako Nakaguchi, Hideo Jinnou, Naoko Kaneko, Masato Sawada, Takao Hikita, Shinji Saitoh, Yasuhiko Tabata, Kazunobu Sawamoto

**Affiliations:** ^1^Department of Developmental and Regenerative Biology, Nagoya City University Graduate School of Medical Sciences, Aichi 467-8601, Nagoya, Japan; ^2^Department of Neonatology and Pediatrics, Nagoya City University Graduate School of Medical Sciences, Aichi 467-8601, Nagoya, Japan; ^3^Department of Biomaterials, Institute for Frontier Medical Sciences, Kyoto University, Kyoto 606-8507, Japan

## Abstract

Recent studies have shown that new neurons are continuously generated by endogenous neural stem cells in the subventricular zone (SVZ) of the adult mammalian brain. Some of these new neurons migrate to injured brain tissues and differentiate into mature neurons, suggesting that such new neurons may be able to replace neurons lost to degenerative disease or injury and improve or repair neurological deficits. Here, we tested whether delivering growth factors via gelatin hydrogel microspheres would support neurogenesis in the SVZ. Insulin-like growth factor-1 (IGF-1)-containing microspheres increased the number of new neurons in the SVZ. Hepatocyte growth factor (HGF)-containing microspheres increased the number of new neurons migrating from the SVZ towards the injured striatum in a stroke model in mouse. These results suggest that the strategy of using gelatin hydrogel microspheres to achieve the sustained release of growth factors holds promise for the clinical regeneration of damaged brain tissues from endogenous neural stem cells in the adult SVZ.

## 1. Introduction

Neural stem cells (NSCs) reside in the brain of adult animals, including humans [1–6]. NSCs residing in the subventricular zone (SVZ) at the lateral walls of the lateral ventricle and in the subgranular zone (SGZ) in the dentate gyrus of the hippocampus produce new neurons and glial cells throughout adult life in mammals [7–11]. The NSCs in the SVZ have the potential to regenerate lost neurons and glia in response to various pathological conditions [12–17]. Neuroregenerative therapy using endogenous NSCs in the SVZ is a highly anticipated emerging strategy for treating human brain diseases because it avoids the risk of immunological incompatibility and the ethical problems inherent in harvesting human cells, and it may reduce the risk of tumorigenesis—which are all problems associated with transplanted stem cells [18, 19]. However, the spontaneous regeneration that takes place in the injured brain is insufficient for its structural or functional restoration [12, 17]. For future clinical applications, the ability to regulate each step in neuronal regeneration, including the generation, migration, differentiation, survival, and functional maturation of new neurons to promote efficient regeneration will be crucial for developing novel and reliable neuronal self-repair strategies [9, 20–23]. 

Various proteins, including neurotrophic factors and paracrine signaling molecules, are reported to enhance neurogenesis in the SVZ [24]. However, one limitation of using these factors in the treatment of brain diseases is the lack of appropriate delivery systems. It is difficult to engineer systemically administered proteins to cross the blood-brain barrier, and such proteins can cause systemic toxicity at high concentrations [25, 26]. On the other hand, a single local injection of liquid drugs into the brain parenchyma may not enhance neuronal regeneration effectively, given the limited volume and persistence of substances administered in this way. Therefore, for clinical applications, safe and effective methods for the sustained delivery of neurogenesis-enhancing factors to the SVZ or injured neural tissues must be developed. 

Here, we report that growth factors released from biodegradable gelatin hydrogel microspheres increase new neurons in the adult mouse brain. Gelatin hydrogels consist of gelatin polymers, which can be electrically complexed with growth factors [27, 28]. They have been used clinically to deliver growth factors in the treatment of patients with diseases including sudden sensorineural hearing loss, Bell's palsy, and peripheral artery diseases [29–32]. In this paper, we tested the effects of insulin-like growth factor (IGF) [33, 34] and hepatocyte growth factor (HGF) [35–37] delivered by gelatin hydrogel microspheres on neurogenesis in the SVZ in the normal brain, and then on the recruitment of SVZ-derived new neurons to the injured brain after stroke in a mouse model.

## 2. Materials and Methods

### 2.1. Preparation of Gelatin Hydrogel

Gelatin hydrogel microspheres (MedGel P15; MedGel, Osaka, Japan) with a diameter within 30 *μ*m were prepared as described previously [27, 28]. The microspheres were incubated with phosphate buffered saline (PBS) (control) or PBS containing recombinant human IGF-1 (PeproTech, Rocky Hill, USA; 0.25 *μ*g) or recombinant human HGF (PeproTech, Rocky Hill, USA; 0.5 *μ*g) for 1 hour at room temperature.

### 2.2. Animals

Adult (8 weeks old) male ICR mice were purchased from SLC (Shizuoka, Japan) and maintained on a 12-hour light/dark cycle with unlimited access to food and water. All animal-related procedures were approved by the Laboratory Animal Care and Use Committee of Nagoya City University.

### 2.3. Injection of Gelatin Hydrogel

In intact mice, 3 *μ*L of the gelatin hydrogel suspension or 2 *μ*L of the solutions without gelatin hydrogel were stereotaxically injected using a capillary micropipette (Drummond Scientific Company, Broomall, PA, USA) into the striatum of anaesthetized 8-week-old ICR mice at the following position relative to bregma: 1.0 mm anterior, 1.5 mm lateral, and 3.2 mm deep (*n* = 5 and 3 animals for groups treated with and without gelatin hydrogel, resp.). In the MCAO model, 11 days after MCAO, 5 *μ*L of gelatin hydrogel suspension or 2 *μ*L of the solutions without gelatin hydrogel were stereotaxically injected using a capillary micropipette into the striatum of anaesthetized 8-week-old ICR mice at the following position relative to bregma: 1.0 mm anterior, 2.0 mm lateral, and 3.2 mm deep (*n* = 6 and 4 animals for groups treated with and without gelatin hydrogel, resp.). After surgery, the animals were left on a heat mat and constantly monitored until recovery. After 7 days, the mice were killed and their brains were prepared for immunohistochemistry. 

### 2.4. Middle Cerebral Artery Occlusion

Middle cerebral artery occlusion (MCAO) was accomplished using the previously described intraluminal filament technique [17, 38, 39]. A laser-Doppler flowmeter probe (model ALF21; Advance, Tokyo, Japan) was attached to the surface of the skull to monitor the regional cerebral blood flow. A silicone-coated 8-0 filament was inserted into the internal carotid artery through an incision in the external carotid artery and then advanced to occlude the middle cerebral artery (MCA). The occlusion of the MCA was confirmed by observing a reduction in the value of laser-Doppler flowmetry of about 30%. The filament was withdrawn 50–60 minutes later, and reperfusion was confirmed by laser-Doppler flowmetry, and the incision was then closed. Gelatin hydrogel microspheres were injected 11 days after MCAO. Seven days after the injection, the mice were killed and their brains were prepared for immunohistochemistry.

### 2.5. Immunohistochemistry and Quantification

Brains were perfusion-fixed with 4% paraformaldehyde, postfixed in the same fixative overnight, and 50 *μ*m sections were cut on a Vibratome sectioning system (VT1200S; Leica, Heidelberg, Germany) as described previously [17, 38, 39]. After three rinses in PBS, the sections were incubated for 40 min in blocking solution (PBS containing 10% donkey serum and 0.2% Triton X-100) and then overnight at 4°C with primary antibodies, which were diluted in the same solution. The next day, the sections were incubated for 2 hours at room temperature with biotinylated secondary antibodies (1 : 500) (Jackson, West Grove, PA, USA) or Alexa Fluor-conjugated secondary antibodies (1 : 500) (Invitrogen, Carlsbad, CA, USA), unless otherwise noted. Biotinylated antibodies were visualized using the Vectastain Elite ABC kit (Vector Laboratories, Burlingame, CA, USA) and DAB (diaminobenzidine tetrahydrochloride).

The primary antibodies (final dilution and source) used in this study were goat anti-doublecortin (anti-DCX) (1 : 100; Santa Cruz Biotechnology, Santa Cruz, CA, USA), rabbit anti-Ki67 (1 : 200; Novocastra, Newcastle, UK), and mouse anti-NeuN (1 : 100; Merck Millipore, Billerica, MA, USA).

For laser scanning microscopy, we used an LSM700 Microscope (Carl Zeiss, Oberkochen, Germany). For the quantification of immunolabeled cells, the images of stained cells were acquired using a fluorescence microscope, BX51 (Olympus, Tokyo, Japan), and a CCD camera, DP71 (Olympus). The actual number of DCX+ cells was counted in three sections: the one containing the injection site and two sections taken 600 *μ*m and 1200 *μ*m, respectively, anterior to the injection site. For analyses of the brain after MCAO, the section including the injection site and an additional 5 (DCX) and 2 (Ki67) anterior sections spaced 300 *μ*m apart were used. The total cell number was estimated by multiplying the sum of the counted cells by 12 and 6, respectively. 

### 2.6. Infarct Volume Evaluation

 Brain sections prepared after MCAO (thickness, 50 *μ*m) were immunostained for NeuN and visualized with DAB using a standard procedure. The images of the sections including the injection site and two additional anterior sections spaced 600 *μ*m apart were captured using a fluorescence microscope, BX51 (Olympus, Tokyo, Japan), and a CCD camera, DP71 (Olympus). 

 For this analysis, we used only mice in which the striatal infarction (NeuN negative area) was observed within 1 mm of the SVZ in at least one section. The areas that lacked NeuN immunoreactivity (infarct area) and those of the ipsilateral hemisphere in the three sections were measured using the Photoshop CS 8.0.1 (Adobe Systems, San Jose, CA, USA) image software. The percentage of the infarct area was determined by dividing the infarct area by that of the ipsilateral hemisphere for each mouse and used for the analysis of infarct volume. 

### 2.7. Statistics

All data are presented as the mean ± SEM. Comparisons between experimental groups were analyzed by two-tailed Student's *t*-test, and differences were regarded as statistically significant when *P* < 0.05.

## 3. Results and Discussion

### 3.1. Study Design

**Figure 1:**
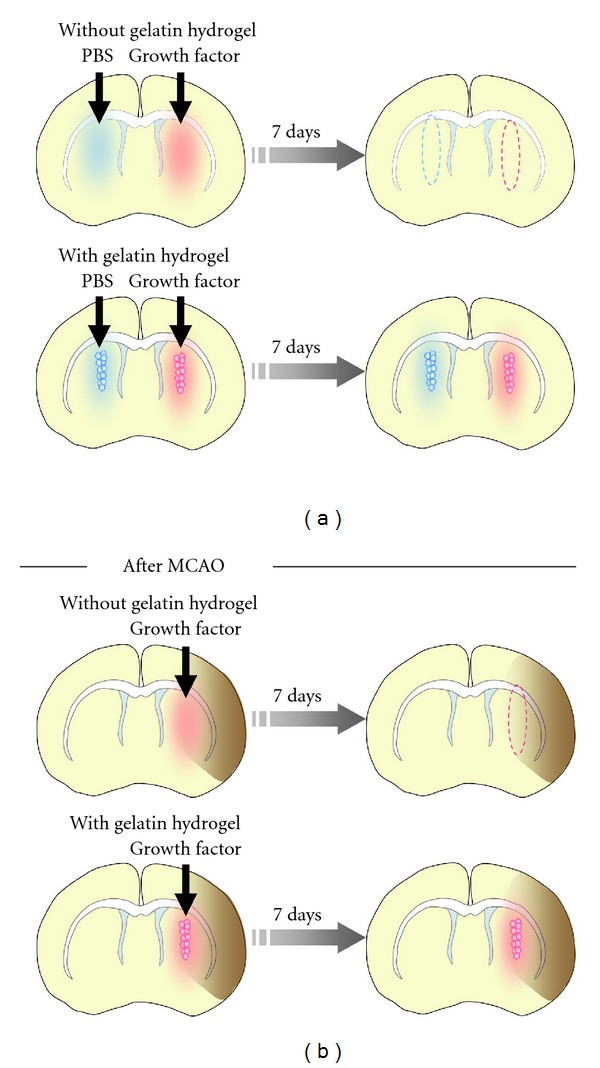
Study design. Schematic illustration of experiments performed to test the effects of growth factors released from gelatin hydrogel on neurogenesis in the SVZ. Effects of growth factors injected into normal (a) and injured brains 11 days after MCAO (b) with or without gelatin hydrogel on the number of new neurons in the SVZ and injured striatum, respectively, were compared 7 days after the injections. Blue and pink circles represent gelatin hydrogel microspheres.

In this study, we focused on two growth factors, IGF-1 and HGF, incorporated into gelatin hydrogel, which can slowly release proteins after implantation into the body [27, 28]; both IGF-1 and HGF have been approved for clinical applications in the treatment of various diseases [29–32, 40–42]. The gelatin hydrogel microspheres were injected into the striatum of normal brain or close to the injured tissue of a stroke model (Figure 1). Seven days after the injection, the brains were fixed and number of DCX+ new neurons was compared. 

### 3.2. IGF

IGF-1 promotes the proliferation of neural stem or progenitor cells as well as neuronal differentiation and survival [43–46]. It has a neuroprotective effect in animal models of stroke [47]. IGF-1-containing gelatin hydrogel microspheres have been used in a clinical trial for the treatment of patients with glucocorticoid-resistant sudden sensorineural hearing loss [29]. However, it is unknown whether the controlled release of IGF-1 using gelatin hydrogel is useful to stimulate neurogenesis in the SVZ.

**Figure 2:**
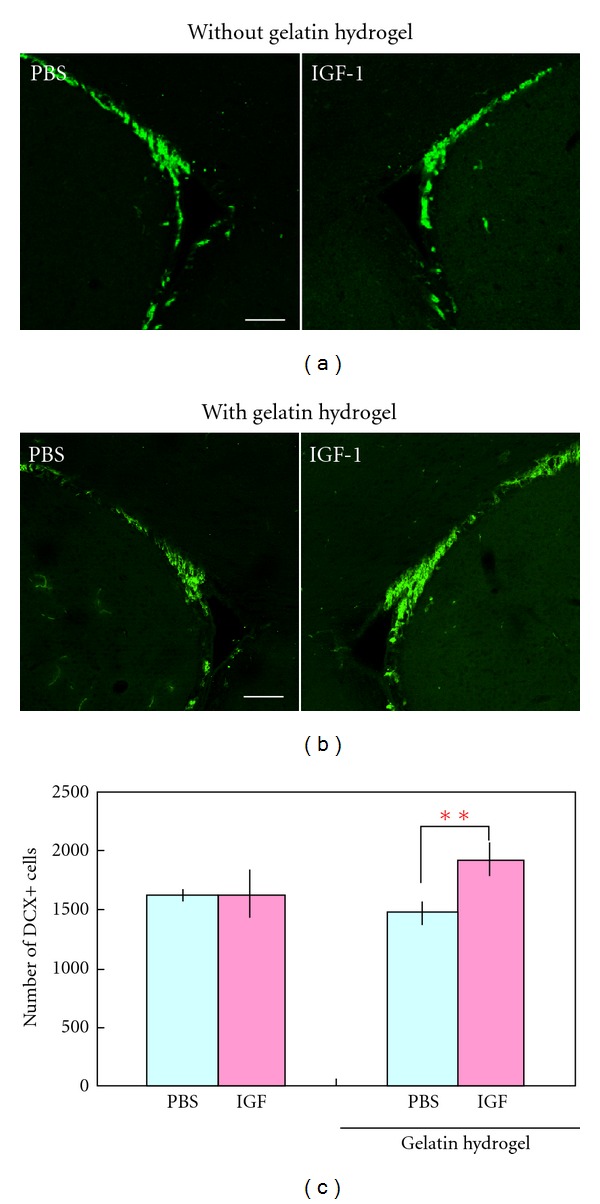
Effects of IGF-1-containing gelatin hydrogel on neurogenesis in the SVZ. (a) Coronal sections of brains that received an IGF-1 or PBS injection without gelatin hydrogel 7 days before, showing DCX+ new neurons in the SVZ (green) (*n* = 3 animals for each group). (b) Coronal sections of brains that received an injection of microspheres containing IGF-1 or microspheres plus PBS, showing DCX+ new neurons in the SVZ (green) (*n* = 5 animals for each group). (c) Quantification of DCX+ cells in the SVZ. Scale bars: 100 *μ*m (a and b). **P < 0.01.

We tested the effects of gelatin hydrogel containing IGF-1 on the number of new neurons in the intact adult mouse SVZ. First, we injected PBS containing IGF-1 (0.25 *μ*g) without hydrogel into the striatum close to the SVZ (Figure 2(a)). Seven days later, there was no significant difference in the numbers of DCX+ new neurons in the SVZ between the IGF-1-injected and PBS-injected control brains (Figure 2(c), PBS: 1621 ± 50 cells, IGF-1: 1633 ± 205 cells, *n* = 3 animals for each group), indicating that the IGF-1 injection without gelatin hydrogel was not effective in stimulating neurogenesis under these experimental conditions. We then injected a gelatin-hydrogel suspension in PBS, with or without the same amount of IGF-1, into the striatum (Figure 2(b)). Seven days later, significantly increased numbers of DCX+ new neurons were observed in the SVZ of brains that received the IGF-1-containing gelatin hydrogel compared with the control group (Figures 2(b) and 2(c), PBS: 1469 ± 99 cells, IGF-1: 1916 ± 143 cells, *n* = 5 animals for each group). These results indicate that gelatin hydrogel is suitable as a vehicle for the delivery of IGF-1 to the SVZ. In addition, these results are consistent with the sustained release of IGF-1 from gelatin hydrogel efficiently promoting IGF-1's actions on the proliferation and differentiation of neural stem or progenitor cells [43–46], resulting in increased numbers of new neurons in the SVZ. 

### 3.3. HGF

Previous studies indicated that HGF stimulates the proliferation of neural stem or progenitor cells in the adult SVZ, as well as the migration and differentiation of new neurons [48–52]. Acute injection of HGF into the adult striatum has a neuroprotective effect and promotes neurological recovery in a mouse model of stroke [53]. Topical application of HGF-containing gels on the surface of the cerebral cortex increases the number of new migrating neurons in the striatum of the MCAO model [54]. HGF-containing gelatin hydrogel has been used for the treatment of animal models of several diseases including noise-induced hearing loss [55] and collagen-induced arthritis [56]. These studies strongly suggest that HGF-containing gelatin hydrogel is likely to be useful to enhance neuronal regeneration after stroke.

We first tested the effects of HGF in PBS, with or without gelatin hydrogel, injected into the striatum, on the number of new neurons in the SVZ of the normal adult brain. Seven days after the injection, there was no significant difference in the number of DCX+ cells between the HGF and PBS-control groups regardless of the use of gelatin hydrogel: with gelatin hydrogel, animals treated with PBS showed 1790 ± 102 new cells, and those given HGF generated 1750 ± 106 cells (*n* = 3 animals for each group); without gelatin hydrogel, animals treated with PBS showed 2111 ± 82 cells; those treated with HGF generated 1992 ± 78 cells (*n* = 5 animals for each group, *t*-test). 

**Figure 3:**
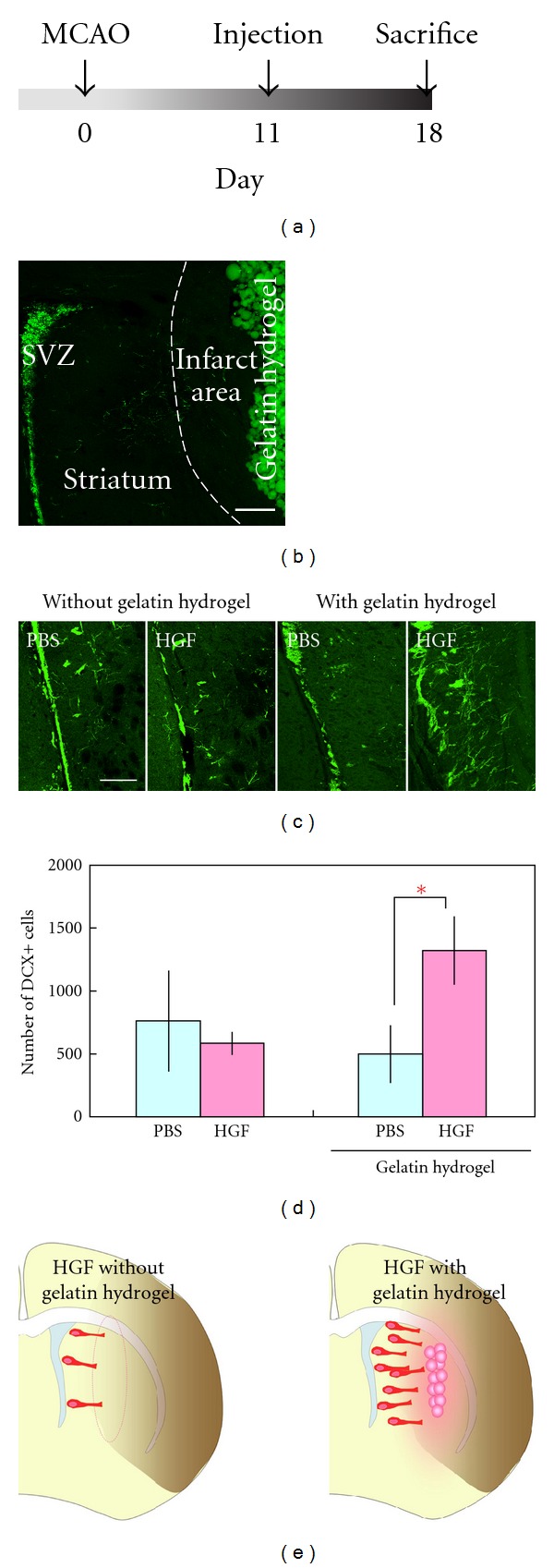
Effects of HGF-containing gelatin hydrogel on the number of new neurons migrating in the ischemic striatum after middle cerebral artery occlusion. (a) Experimental design. (b) A coronal brain section showing DCX+ new neurons in the SVZ (green) and gelatin hydrogel that was injected into the striatum. (c) Coronal sections stained for DCX (green) from brains that received a PBS or HGF injection with (*n* = 6 animals for each group) or without (*n* = 4 animals for each group) gelatin hydrogel. (d) Quantification of the number of DCX+ cells separated by at least 50 *μ*m from the SVZ in the ipsilateral striatum. (e) Summary of the results. HGF administered as HGF-containing gelatin hydrogel microspheres significantly increased the number of new neurons. Scale bars: 200 *μ*m (b and c). *P < 0.05.

Next, we tested the effects of injecting the same substances in a mouse model of stroke induced by MCAO. In this model, SVZ-derived new neurons can be found migrating toward the injured striatum 2-3 weeks after the induction of ischemia [17]. Therefore, we injected HGF in PBS, with or without gelatin hydrogel, into the striatum 11 days after MCAO (Figures 3(a) and 3(b)). Eighteen days after MCAO, we quantified the infarct volume and number of new neurons in the striatum. The HGF administration did not affect the infarct volume, regardless of the use of gelatin hydrogel microspheres: with gelatin hydrogel, PBS yielded an infarct volume of 26.3 ± 3.4%, and those treated with HGF had an infarct volume of 25.3 ± 2.0% (*n* = 6 animals for each group); without gelatin hydrogel, animals treated with PBS showed an infarct volume of 26.6 ± 1.6% and those treated with HGF had an infarct volume of 29.6 ± 3.6% (*n* = 4 animals, *t*-test). 

The administration of HGF without gelatin hydrogel also did not affect the number of DCX+ cells in the striatum (Figures 3(c) and 3(d)). However, the HGF-containing gelatin hydrogel significantly increased the number of DCX+ cells migrating in the striatum after MCAO (Figures 3(c) and 3(d)). Interestingly, the majority of the DCX+ cells extended a long leading process toward the injected gelatin hydrogel, suggesting that these cells were migrating laterally. Because HGF has been reported to stimulate the proliferation of SVZ progenitors [48], we also examined the effects of HGF on cell proliferation in the SVZ after MCAO. There was no significant difference in the number of Ki67+ proliferating cells between the HGF and PBS-control groups, regardless of the use of gelatin hydrogel (with gelatin hydrogel, PBS: 796.5 ± 87.65 cells, HGF: 798.8 ± 96.12 cells, *n* = 6 and 5 animals, respectively; without gelatin hydrogel, PBS: 837.5 ± 78.77 cells, HGF: 926.0 ± 109.4 cells, *n* = 4 animals for each group, *t*-test). These results suggest that the increased number of new neurons in the injured striatum induced by treatment with HGF with gelatin hydrogel was due to an increased efficiency of the new neurons' migration but not to their production in the SVZ (Figure 3(e)).

Since the receptor for HGF, c-Met, is expressed in adult DCX+ migrating neurons [49] as well as GFAP+ astrocytes [50], it is possible that DCX+ cells are attracted by the HGF-releasing gelatin hydrogel. It is also possible that the HGF-induced increase in the number of DCX+ cells in the striatum was caused by angiogenic activity [54], since new neurons use blood vessels for their migration [17, 38].

## 4. Conclusions

The benefits reported here of using gelatin hydrogel microspheres to deliver growth factors to simulate neurogenesis in the SVZ demonstrate that gelatin hydrogel is a promising vehicle for the local and sustained release of drugs in the brain. Previous studies demonstrated that the epicortical delivery of EPO [57] and HGF [54] enhance neurogenesis in the SVZ in mouse models of stroke. However, considering the longer distance between the brain surface and the SVZ in the human brain, direct injection inside the brain parenchyma may be needed to efficiently stimulate neurogenesis from neural stem cells in the SVZ. The local delivery of drugs using hydrogels should result in a lower total dose of medications and thus in fewer side effects. In addition, this technology should be applicable to any charged protein that can enhance neural regeneration [58]. Further studies are needed to improve the injectability of the gelatin hydrogel, which will result in a less invasive procedure. Finally, how the newly generated neurons produced by this treatment contribute to neurological improvement needs to be elucidated.
